# SpatialPPIv2: Enhancing protein–protein interaction prediction through graph neural networks with protein language models

**DOI:** 10.1016/j.csbj.2025.01.022

**Published:** 2025-01-23

**Authors:** Wenxing Hu, Masahito Ohue

**Affiliations:** School of Computing, Institute of Science Tokyo, Yokohama, Kanagawa, Japan

**Keywords:** Protein-protein interaction, Deep neural network, Graph attention network

## Abstract

Protein–protein interactions (PPIs) are fundamental to cellular functions, and accurately predicting such interactions is crucial for understanding biological mechanisms and facilitating drug discovery. SpatialPPIv2 is an advanced graph-neural-network-based model that predicts PPIs using large language models to embed sequence features and graph attention networks to capture structural information. By leveraging the comprehensive PINDER dataset, which includes interaction data from the RCSB PDB and AlphaFold databases, SpatialPPIv2 improves the PPI prediction specificity and robustness. Unlike the original SpatialPPI, the updated version no longer depends on protein structure prediction algorithms and can predict protein interactions standalone. SpatialPPIv2 outperforms the state-of-the-art PPI predictors, demonstrating superior accuracy and reliability. Furthermore, the model was robust when using structural prediction methods, including AlphaFold3, AlphaFold2, and ESMFold, indicating its applicability even if experimentally determined structures are unavailable. SpatialPPIv2 offers a promising solution for accurately predicting PPIs, provides insight into protein function, and supports advances in drug discovery and synthetic biology. SpatialPPIv2 is available at https://github.com/ohuelab/SpatialPPIv2.

## Highlights

• SpatialPPIv2 offers a promising solution for accurately predicting PPIs.
• SpatialPPIv2 outperforms the state-of-the-art PPI predictors.
• Useful, even if experimentally determined structures are unavailable.

## Graphical abstract

**Figure:**
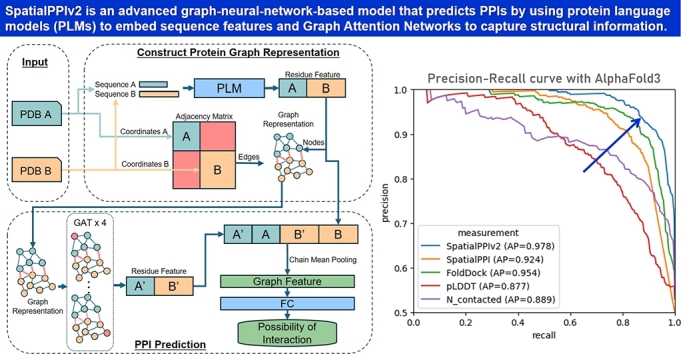

## 1 Introduction

Protein–protein interactions (PPIs) are fundamental to nearly all biological processes, including signal transduction, metabolic regulation, and cellular architecture. Understanding these interactions is essential for elucidating the molecular mechanisms of life, annotating protein functions, and identifying potential therapeutic targets. However, experimentally determining PPIs is labor-intensive, costly, and scale-limited, necessitating the development of computational approaches for accurate and efficient PPI predictions [1], [2], [3].

PPI prediction can be broadly categorized into sequence- and structure-based approaches. Sequence-based methods are widely used owing to the abundance of protein sequence data and their computational efficiency [4], [5], [6]. Sequence-based approaches such as D-script [7] and Topsy-Turvy [8] exploit amino acid sequences and evolutionary information to predict interactions. D-script leverages neural networks to represent and predict PPIs by learning sequence patterns, whereas Topsy-Turvy uses transfer-learning techniques to enhance prediction performance based on sequence data. However, sequence-based approaches lack spatial information of proteins, which is crucial for determining their physical interactions [9]. This often restricts their ability to accurately distinguish between interacting and non-interacting proteins. In contrast, structure-based PPI prediction methods incorporate the three-dimensional (3D) structural information of proteins, providing a direct representation of their potential interactions [10], [11]. Structure-based methods predict the physical compatibility of binding surfaces and identify specific contact regions, which is an advantage over sequence-based approaches [12], [13]. Struct2Graph [14] and the GNN-PPI [15] are notable structure-based prediction methods. Struct2Graph converts 3D protein structures into graphs and applies graph-based learning to infer interactions. GNN-PPI also uses graph neural networks to capture both local and global protein structures. Although structure-based predictions offer specificity for identifying binding interfaces, their applicability is often hindered by the scarcity of experimentally determined protein structures and the computational expense associated with structural data processing [16]. Despite these challenges, structure-based methods provide interpretability, which makes them desirable when structural data are available. Consequently, we adopted a structure-based prediction strategy in this study.

Recent advances in protein structure prediction have provided valuable solutions that address the scarcity of protein structural data. AlphaFold2 [17], developed by DeepMind, is a ground-breaking artificial intelligence (AI) model for protein structure prediction. AlphaFold2 leverages a combination of attention mechanisms and end-to-end differentiable networks to predict 3D protein structures from amino acid sequences with atomic-level accuracy. AlphaFold2 won the CASP14 competition [18] for its unprecedented ability to solve the protein-folding problems. AlphaFold3 [19] represents the next iteration of DeepMind's AlphaFold series, further improving PPI predictions as well as multichain assemblies. AlphaFold3 integrates advanced graph neural networks and optimizations in large-scale protein complex modeling, addresses the limitations of multi-chain predictions, and offers enhanced scalability for larger biological systems. ESMFold [20], which was introduced by Meta-AI, is a protein structure prediction model that leverages large-scale protein language models trained using extensive sequence data. Unlike traditional methods, ESMFold does not rely on external sequence alignments or structural template databases, thereby enabling rapid structural prediction directly from a single protein sequence. This approach emphasizes speed and scalability by integrating pretrained sequence representations with lightweight structural modeling, making it highly suitable for high-throughput and large-scale applications. These methods collectively advance the field of structural biology by addressing diverse challenges in protein modeling and functional prediction.

The FoldDock [21] algorithm and SpatialPPI [22] use protein complex prediction methods. FoldDock is a computational model designed to predict PPIs using the pDockQ score calculated from AlphaFold2 predictions. FoldDock uses the average interface pLDDT score and number of contacted residues of the predicted complex to calculate pDockQ scores using a sigmoidal fit. pDockQ score calculation is feasible for different structure prediction programs. SpatialPPI leverages deep neural networks to analyze protein complexes predicted by the AlphaFold Multimer [23] to predict interactions. This is achieved by converting the atomic coordinates into spatial models and computing the atomic distributions to capture the structural organization of the complexes. By using image-processing techniques, SpatialPPI can effectively extract essential 3D structural features, enabling accurate and robust prediction of protein interactions.

The Protein Interaction Dataset and Evaluation Resource (PINDER) dataset [24] provides a comprehensive collection of PPIs and docking data. Compared to other state-of-the-art datasets, such as DIPS-Plus [25], ProteinFlow [26], and PPIRef [27], PINDER contains more than 500 times more data and offers greater diversity in training flexible models capable of adapting to predicted structures. PINDER includes structural data from both the RCSB Protein Data Bank [28] and the AlphaFold Database [29], using Interface-Based Clustering and Deleaking techniques to ensure high-quality, non-redundant data. The large size and quality of the PINDER dataset makes it an excellent resource for training models to predict both protein interfaces and overall interactions, thereby enhancing model robustness and generalizability. The training set of PINDER comprises millions of dimers and offers diverse examples that facilitate the capability of models to accurately capture different types of interactions, including challenging cases involving diverse protein structures.

Reliable negative data are essential for the development and evaluation of PPI prediction models [30], [31]. Negatome 2.0 [32] is a curated dataset specifically designed to provide high-confidence, non-interacting protein pairs. Negatome 2.0 uses rigorous filtering criteria based on experimental evidence and biological knowledge to ensure that the included protein pairs are unlikely to interact. This dataset serves as a valuable resource for benchmarking computational methods because it minimizes the risk of false negatives that often arise from unobserved interactions in other datasets. However, the non-interacting data included in Negatome 2.0 is limited. Generating negative-label data from failed experiments is relatively difficult. Since the failure to identify PPI in most biological experiments indicates that PPIs are not observed under these experimental conditions, proving that the two proteins have never interacted is difficult. Considering that only 0.325–1.5% of protein pairs have PPI [33], the computational method uses randomly sampled protein pairs as negative-label data, most of which are likely to be truly non-interacting. Therefore, random sampling is acceptable for training models using large-scale interaction datasets [16].

In the field of protein representation learning, transformer-based protein language models have emerged as powerful tools for capturing meaningful sequence embeddings, enabling significant advancements in downstream tasks, such as structure prediction, function annotation, and PPI analysis. Among these, ProtBert and ProtT5 [34], inspired by natural language processing models, represent major milestones. ProtBert adapts the BERT architecture [35] to protein sequences and trains it on large-scale datasets to learn contextualized embeddings that capture the semantic and functional properties of amino acid sequences. Similarly, ProtT5 extends the transformer architecture with sequence-to-sequence capabilities, enabling fine-tuned performance in diverse protein-related tasks, including sequence generation and mutation prediction. ESM-2 [20], [36] sets a new benchmark for protein sequence modeling by using large-scale transformer architectures trained on billions of protein sequences. ESM-2 demonstrates improved representation learning and enables high-resolution tasks such as structure prediction and evolutionary modeling to achieve state-of-the-art performance in a range of applications. ESM-2 focuses on scaling both the model size and training data, pushing the boundaries of protein language modeling and opening new possibilities for understanding and engineering proteins. These advancements highlight the growing impact of deep learning in computational biology, paving the way for accurate and scalable methods to study protein properties and interactions [37], [38].

Here, we present SpatialPPIv2, a novel structure-based PPI prediction framework, to address the challenges discussed above. SpatialPPIv2 uses language model-based embedding to obtain residue sequence information and captures the residue relationship between two proteins through the attention mechanism of a graph neural network, thereby achieving excellent performance. Compared to the original SpatialPPI, SpatialPPIv2 no longer relies on structural prediction methods and can work alone. We also verified the model performance when used with ESM-2 contact prediction and used the predicted structures from AlphaFold3, AlphaFold2, and ESMFold to ensure that SpatialPPIv2 remains reliable when there are no available structural data. In addition, we verified the versatility of SpatialPPIv2 on large-scale datasets and discussed the inference process of SpatialPPIv2 through visualization. This study demonstrates the utility of SpatialPPIv2 in effectively predicting PPIs and highlights its application in studying interactions, thereby accelerating research in the fields of drug discovery and synthetic biology by providing a method to rapidly screen interacting protein pairs.

## 2 Materials and methods

**Fig. 1:**
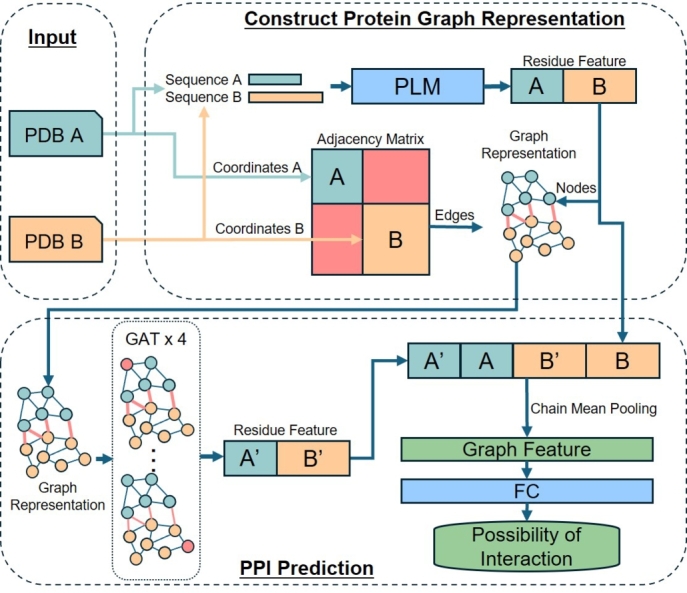
Workflow of SpatialPPIv2. Input protein structures (PDB files) were used to extract structural and sequence features. The distance matrix within proteins was used to construct the edges in the protein graph representation. The protein sequence information encoded by the protein language model (PLM) was used as the features of nodes in the graph representation. The distance matrix within proteins was used to construct the edges in the protein graph representation. The protein sequence information encoded by language models was used as the node features in the graph representation. The residues between two proteins are fully connected to increase message passing between proteins. The overall graph features of protein pairs were obtained by chain mean pooling the protein features directly obtained from language models and the relationship features output by GAT and were used to calculate the possibility of interaction between proteins.

SpatialPPIv2 is a graph neural network-based deep learning model designed to predict PPIs using both structural and sequence information. The workflow is shown in Fig. 1. Protein structure files (PDB) were processed to extract features, including amino acid sequence features and adjacency matrices, to construct graph representations of the proteins. A fully connected graph was constructed between each residue pair from the two proteins to extract the relationship features between the proteins. By concatenating the features of each protein directly obtained from the language models and the relationship features between protein outputs by GAT, chain mean pooling was used to extract the overall graph features of the protein pair. Finally, the possibility of an interaction between the two proteins was output through a fully connected layer.

### 2.1 Dataset construction

Constructing a high-quality dataset is crucial for the performance and generalizability of deep neural network models. The PINDER database provides a comprehensive collection of PPIs and docking data. PINDER includes structural data from both the RCSB PDB and the AlphaFold database and uses interface-based clustering and deleaking. Two interfaces were marked as similar if they met the following criteria: iRMS <5.0, IS-score >0.3, and log(P-value) <−9.0, where iRMS is the interface RMSD after alignment, the IS-score represents the interface similarity score, and log(P-value) is the logarithm of the statistical significance of the IS-score determined from the distribution of interface scores using the iAlign methodology [39]. Based on the clustering results, the dataset was divided into training, validation, and test sets, with redundancy removed and filtering applied to ensure the highest quality. The PINDER training set consisted of 1,560,682 dimers from 42,220 clusters, of which 136,498 had paired apo structures and 566,171 had paired AlphaFold Database structures. The dataset was further filtered for our purposes. Pairs of residues with a C_*α*_ distance of less than 8 Å were considered to be in contact [40]. Only residue pairs with more than eight contacts and protein sequence lengths between 35 and 300 residues were used. The interaction data were used as positive data in the dataset.

Negatome 2.0 served as an important resource for noninteracting protein pairs. Negatome 2.0, which provides manually curated, experimentally verified, noninteracting protein pairs, is reliable for evaluating the PPI prediction performance [41]. Negatome 2.0's manual-stringent dataset contains 1991 pairs of proteins that do not interact. This dataset contains manually annotated literature data describing the lack of protein interactions and was filtered against the IntAct [42] database. As proteins from this dataset lacked experimentally determined protein structure data, we extracted only 168 protein pairs from this set. Moreover, the PDB-stringent dataset was used. The PDB-stringent dataset contained pairs of proteins belonging to at least one structural complex that did not interact directly. A total of 432 non-interacting pairs were selected from this dataset. However, because of the limited availability of experimentally verified negative samples, non-interacting pairs from Negatome 2.0 were used for the evaluation in this study.

The negative training and validation datasets were generated by random sampling to create an equal number of negative and positive samples and were cross-checked with BioGRID [43] for validation. Specifically, two proteins were randomly selected from all proteins in the split positive set as a possible negative pair. The selected possible negative pairs were checked using the BioGRID database to exclude known protein pairs that might interact. The protein pairs examined constitute the negative dataset for training and validation. This approach may have lower quality than experimentally determined non-interacting protein pairs. However, due to the difficulty of experimentally determining non-interacting protein pairs, this method ensures the universality of protein pair selection through large data volumes. Simultaneously, the reliability of the negative data set selected based on random sampling was verified using experimentally determined non-interacting protein pairs from Negatome 2.0 as the test set.

Table 1 summarizes all datasets used in this study and their final filtered sizes. The final dataset comprised 1,167,032 pairs for training, 2,162 pairs for validation, and 1,200 pairs for testing.

**Table 1:** Datasets used in this study.

Usage	Label	Data Source	Number
Train	Positive	Pinder Train	583,516
	Negative	Random Sampling from Train	583,516

Validation	Positive	Pinder Val	1,081
	Negative	Random Sampling from Val	1,081

Test	Positive	Pinder Test	600
	Negative	Negatome 2.0 manual stringent	168
		Negatome 2.0 pdb stringent	432

### 2.2 Sequence feature extraction

Residual sequence features were extracted using language models. Three state-of-the-art protein language models were compared: ProtBert and ProtT5 from ProtTrans and ESM-2 from Evolutionary Scale Modeling (ESM). The details of the model parameters are listed in Table 2. ProtBert [44] was selected because it has been used in several protein-related studies [37], [38], [45]. As an Encoder-only model, ProtBERT has a fast inference time and is easy to deploy. Specifically, we used the ProtT5-XL-UniRef50 model [46] referred to as ProtT5. Because ProtT5 is a sequence-to-sequence model, it is flexible and suitable for various downstream tasks. For the ESM-2 language model, we used a 33-layer model with 650 million parameters. This is because, for larger models, their embedding dimensions also increase significantly, resulting in changes in the number of network parameters that significantly increase the computational difficulty. Therefore, we chose the esm2_t33_650M_UR50D model [47], which is similar to the embedding dimensions of ProtBERT and ProtT5. All three models were masked language models and were trained using unsupervised learning without human-labeled data.

**Table 2:** Details of Protein Language Models.

Full Name	Dataset	Layers	Params	Dim
ProtT5-XL-UniRef50	UniRef50	24	3B	1,024
ProtBert	UniRef100	30	420M	1,024
esm2_t33_650M_UR50D	UniRef50	33	650M	1,280

A one-hot encoder was implemented as a baseline to indicate the importance of the language models. Each residue was transformed into an *L*-dimensional vector representation, where *L* is the embedding dimension of the language model. Using language models significantly improves the processing speed compared with database-based methods, such as retrieving embeddings from UniRef [48]. Moreover, language models exhibit a superior performance in predicting novel or designed proteins that lack homologous structures [49].

### 2.3 Graph representation of protein

**Fig. 2:**
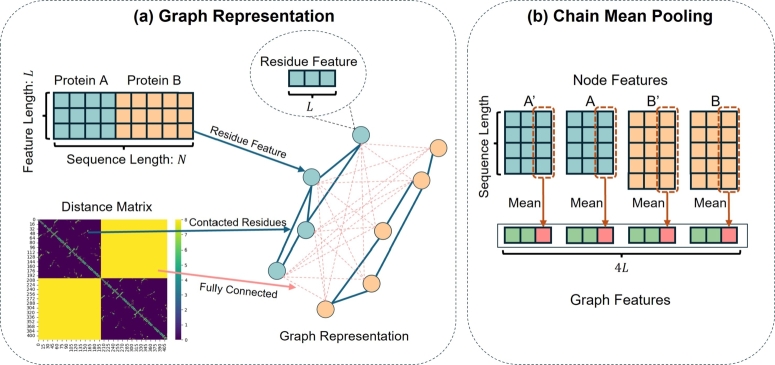
Data Flow in SpatialPPIv2. (a) The distance matrix is computed from the combined distances between residues in the same proteins. Edges are added between residues in the different proteins to form a fully connected structure. Each node in the graph represents a residue feature vector generated by language models. (b) Chain mean pooling applied on residue features A and B from both language model outputs and node features A' and B' from GAT outputs to generate the graph feature vector.

The input protein pairs were transformed into graphical representations, as shown in Fig. 2(a). Each residue in the protein was represented as a node in the graph, with features computed using language models. The C_*α*_ distance between residues in the same protein was used to compute the distance matrix. An edge was constructed between two residual nodes if the C_*α*_ distance between them was less than 8 Å [40]. The edge weight was set as the distance between residues. A fictional edge was constructed for each pair of residues between the input proteins to enhance message passing between the two structures. The weights of these fictitious connections were set to a distance threshold of 8 Å. Although this dense connection method increases the computational and memory requirements, compared to using a Siamese network to process input data separately [50], this dense connection focuses more on the possible residual relationships between the two proteins.

### 2.4 Network structures

The graph representations of the input proteins were updated using four graph attention (GAT) layers [51]. GAT dynamically adjusts the weights of information propagation between nodes based on their features, allowing different neighbors to have varying levels of influence on a central node, thereby enhancing the performance of the model on complex graph structures. With its attention calculation, as in (1), GAT is suitable for handling heterogeneous node features and intricate dependencies [52].

(1) $$\alpha_{ij}=\frac{\exp\left(\text{LeakyReLU}\left(a^{⊤}\left[Wh_{i}\|Wh_{j}\right]\right)\right)}{\sum_{k\in N_{i}}\exp\left(\text{LeakyReLU}\left(a^{⊤}\left[Wh_{i}\|Wh_{k}\right]\right)\right)}$$

Each GAT layer was set to four attention heads. The multi-head attention was averaged to obtain the output. The number of hidden dimensions forms a U-shape [L/2, L/4, L/2, *L*]. Batch normalization was applied between the GAT layers to stabilize the training process and improve the training speed. SpatialPPIv2 handles the unknown distance relationship between two protein structures by relying on the attention mechanism of the GAT. The fully connected relationship between two proteins generated during the graph representation process was adjusted using this attention mechanism to adjust the message-passing coefficients of the residues between the two proteins. Because the weights are calculated dynamically and depend on features rather than on fixed graph structures, GAT can adapt to the feature distribution on the graph flexibly and is not prone to overfitting because of the structural similarity and simple memory of the protein sequences [53].

The node features from the GAT concatenated with the residue features from the language models were aggregated using chain-mean pooling to generate graph features, as shown in Fig. 2(b). Chain mean pooling involves applying global mean pooling separately to residues from each of the two proteins and then concatenating the resulting features to form a graph representation. The graph features were then passed through a fully connected (FC) layer to predict the likelihood of an interaction between the two input proteins.

GAT mainly analyzes the feature relationship between two proteins and extracts the overall features of the protein pair through the attention mechanism. The residue features direct output from language models containing detailed information from the amino acid sequence. This combination of global semantic information and local information ensures the accuracy of prediction.

In addition, based on the attention-based contact prediction capability of ESM-2 [54], we constructed a fully sequence-based model called ESM-2+ac. The model uses weights trained on ESM-2 residue embedding and the experimental structure but only uses the contact map based on attention contact predictions at inference. The purpose of this model is to make rapid inferences when structural data are unavailable. Specifically, we used the contact prediction of ESM-2 to predict the contact graphs of the two proteins. An edge with a weight of 8 was set between the residues with a contact probability greater than 0.5. The edge between the two proteins uses the same full connection as described above.

Interacting protein pairs were labeled as 1, whereas non-interacting protein pairs were labeled as 0. The model output, representing the interaction probability, was compared with the ground truth labels using the binary cross-entropy loss function to compute the training loss. The model was optimized with the Adam optimizer with a learning rate of 0.0001. Training was conducted over 10 epochs with a batch size of 8. Gradient accumulation was employed across four batches to effectively utilize larger batch sizes without exceeding memory constraints, resulting in an effective batch size of 32 for loss computation.

### 2.5 Evaluations

In this study, we compared the performance of SpatialPPIv2 with state-of-the-art methods including Topsy-Turvy, D-script, Struct2Graph, and GNN-PPI. Topsy-Turvy and D-script were obtained from their official websites and used with the pre-trained datasets for prediction. Struct2Graph and GNN-PPI were obtained from their GitHub repositories, and the data were downloaded and trained according to their reproducibility guidelines. The models were evaluated using key binary classification metrics, including accuracy, precision, recall, F1 score, average precision (AP), and area under the receiver operating characteristic curve (AUROC). These indicators are defined as follows.

(2) $$\text{Accuracy}=\frac{\text{TP}+\text{TN}}{\text{TP}+\text{FP}+\text{FN}+\text{TN}}$$

(3) $$\text{Precision}=\frac{\text{TP}}{\text{TP}+\text{FP}}$$

(4) $$\text{TPR}=\text{Recall}=\frac{\text{TP}}{\text{TP}+\text{FN}}$$

(5) $$\text{FPR}=\frac{\text{FP}}{\text{FP}+\text{TN}}$$

(6) $$\text{F1 Score}=\frac{2\cdot \text{Precision}\cdot \text{Recall}}{\text{Precision}+\text{Recall}}$$

(7) $$\text{AP}=\int_{0}^{1}\text{Precision}(\text{Recall})\;d(\text{Recall})$$

(8) $$\text{AUC}=\int_{0}^{1}\text{TPR}(\text{FPR})\;d(\text{FPR})$$

The structural data for most proteins are not available because of the complexity of protein structure determination. We used three protein structure prediction models, AlphaFold3, AlphaFold 2.3.1, and ESMFold_v1, to predict the test dataset and evaluated the performance of SpatialPPIv2, FoldDock, and SpatialPPI using the predicted structures. We directly used the predicted protein structures to calculate pDockQ using the script officially provided by FoldDock to represent the performance [55]. The pDockQ score for FoldDock was calculated as follows:

(9) $$\text{pDockQ}=\frac{L}{1+e^{-k(x-x_{0})}}+b,$$

where x=interface_pLDDT⋅log⁡(n_contacts), L=0.724, $x_{0}=152.611$, k=0.052, and b=0.018. if_pLDDT is the average pLDDT score of the contact residues and n_contacts is the number of contacted residues.

This test aims to determine how the model performs when structural data are unavailable. In particular, we used the AP to measure the accuracy of individual prediction models and model-based prediction programs. The AP measures the overall precision-recall performance at different thresholds. This is applicable not only to binary prediction methods with output values between zero and one. The AP calculated for the number of contact residues predicted by the structure-prediction model and the average pLDDT score of the interface also indicated the model's discrimination between the input proteins and whether they can interact.

The structural predictions and training of SpatialPPIv2 were executed on the computing nodes from TSUBAME 4.0. Each node contains two AMD Epyc 9654 processors with 768 GB of RAM and four NVIDIA H100 Tensor Core GPUs. With the previously calculated embedding of the training set, each epoch of training SpatialPPIv2 took approximately 3 h and predictions were made at a speed of approximately 120 pairs/s. Including calculating the embedding of sequences, the inference speed of SpatialPPIv2 is 0.5 s per pair on average.

For reference, the predictions of FoldDock and SpatialPPI themselves take negligible time. However, their predictions are based on protein complexes predicted by structure prediction programs. On the same machine, it takes 23 min on average to predict a protein complex using AlphaFold3, whereas AlphaFold2 requires 96 min. Similarly, for Topsy-Turvy, D-script, Struct2Graph, and GNN-PPI, their prediction speeds can reach about one hundred pairs per second. Their main computational time cost is in the pre-processing stage. The average speed of Topsy-Turvy and D-script in the pre-processing stage is three proteins per second. That number is 5 proteins per second for Struct2Graph, whereas the same for GNN-PPI can reach 30 proteins per second due to its more straightforward pre-processing stage.

## 3 Results and discussion

### 3.1 Comparison of residue embeddings

**Fig. 3:**
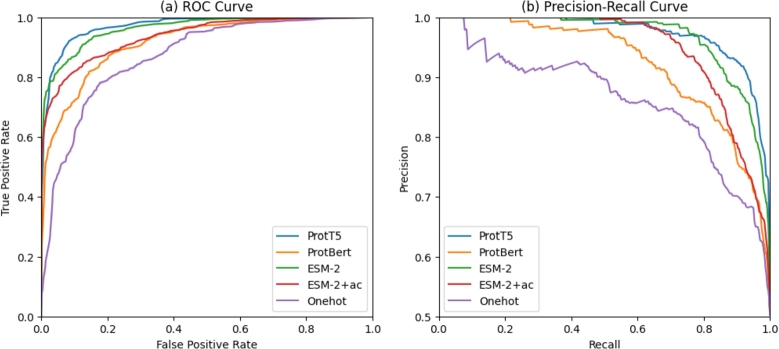
Performance evaluations of different models. (a) Receiver operating characteristic (ROC) curve for different embedding methods of the test dataset. (b) The precision-recall curve of different embedding methods of the test dataset.

The performances of SpatialPPIv2 using ProtT5, ProtBert, ESM-2, one-hot encoding, and the fully sequence-based ESM-2+ac model were tested and compared. Fig. 3(a) shows the receiver operating characteristic (ROC) curve for each model in the test set, which represents the true and false positive rates of the model. The discriminative ability of each model was relatively similar, indicating the accuracy of SpatialPPIv2 in predicting positive results. Fig. 3(b) shows the precision-recall curve of each model. The model using one-hot encoding dropped fast in the upper left interval. This is because the one-hot model does not contain substantial information regarding the residues, but only includes the types of amino acids, which makes the sequence information directly transferred from the embedding to the classifier ineffective. Consequently, negative samples that should have been identified based on residue features cannot be correctly detected and are output as false positives.

Table 3 lists the accuracy, precision, recall, F1 score, average precision (AP), area under the receiver operating characteristic curve (AUROC), and confusion matrix of each model. The results obtained using the ProtT5 language model had the best overall performance, whereas the model using ESM-2 was similar in terms of both AP and AUROC. The ProtT5 language model performed the best overall, whereas the model using ESM-2 was similar in terms of both AP and AUROC. The results of the ProtBERT model were lower than expected, and several false negatives were reported. Compared to the output range of ProtT5 between -1 and 1, the embedding outputs by ProtBert are mostly distributed between -1 and 1 but also have more extreme values greater than 5. This uneven encoding distribution may make it difficult for the neural networks to converge to a suitable range [56]. The ESM-2+ac model also achieved good performance, providing a solution for large-scale reasoning in the absence of suitably structured data.

**Table 3:** Comparison of accuracy (ACC), area under receiver operating characteristic curve (AUROC), average precision (AP), F1 score, precision, recall, and confusion matrix for different embedding methods. One-hot encoding is used as a performance baseline to illustrate the effect of embedding using a protein language model.

	ProtT5	ProtBert	ESM-2	ESM-2+ac	One-hot
Accuracy	0.910	0.823	0.891	0.858	0.762
Precision	0.886	0.861	0.872	0.880	0.716
Recall	0.942	0.772	0.917	0.830	0.868
F1 Score	0.913	0.814	0.894	0.854	0.785
AP	0.973	0.922	0.967	0.947	0.862
AUROC	0.972	0.916	0.963	0.937	0.870

TN	527	525	519	532	393
FP	73	75	81	68	207
FN	35	137	50	102	79
TP	565	463	550	498	521

### 3.2 Predicting with protein structure prediction methods

**Fig. 4:**
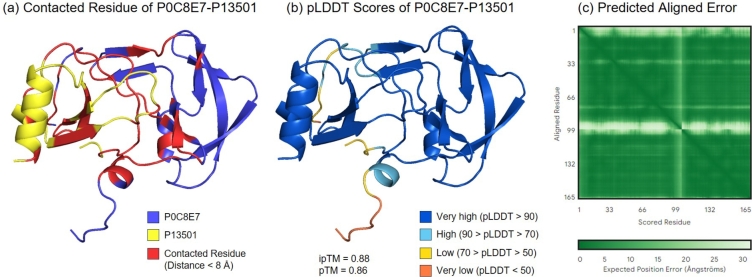
AlphaFold3 predictions for the protein pair P0C8E7 and P13501. These proteins have been experimentally verified to not interact. (a) Contacting residues between the two proteins predicted by AlphaFold3. (b) pLDDT score represents the prediction confidence, with higher scores indicating high confidence in the structure. (c) Predicted aligned error (PAE) estimates the error in the relative position and orientation between residues, with lower PAE values indicating higher confidence in the predicted structure.

AlphaFold does not always successfully determine whether the input proteins can interact. In some cases, even when the input proteins cannot physically interact, AlphaFold produces a tightly bound protein complex. Fig. 4 shows the protein complexes of P0C8E7 and P13501 predicted using AlphaFold3. P0C8E7 and P13501 were experimentally verified to lack interactions, which were manually recorded using Negatome 2.0 [57]. In Fig. 4(a), the blue chain represents the protein P0C8E7 and yellow represents P13501. The red area represents interface residues whose distances from each other are less than 8 Å. Fig. 4(b) shows the pLDDT scores of the predicted structures. Most of the structures, including the contact interface shown in Fig. 4(a) are dark blue, representing AlphaFold3 with high confidence scores. Fig. 4(c) shows the predicted alignment errors (PAE). PAE estimates the error in the relative position and orientation between the two residues in the predicted structure, with higher values indicating greater uncertainty and lower confidence. The two white lines (one horizontal and one vertical) represent the first part of the protein sequence and divide the figure into four areas. The upper right and lower left areas represent the interacting regions between the two protein domains, which AlphaFold3 believes the prediction results have high credibility.

**Fig. 5:**
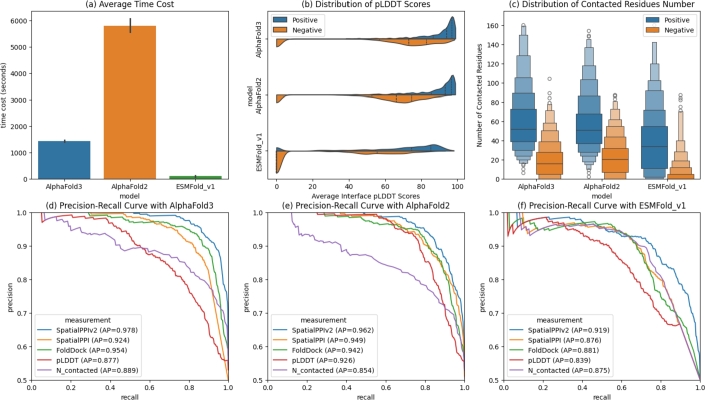
Comparison of protein complex predictions from AlphaFold3, AlphaFold2, and ESMFold_v1. (a) Average prediction time. The prediction time for AlphaFold3 and AlphaFold2 includes the database search. (b) The average interface pLDDT distribution of the complexes predicted by the three models classed by the data label. (c) The distribution of the number of contacted residues of the complexes predicted by the three models classed by the data label. (d) Precision-recall curves of predicting interactions of protein complexes predicted by AlphaFold3 based on SpatialPPIv2, FoldDock, SpatialPPI, and average interface pLDDT score (pLDDT) and number of contacted residues. The values in brackets represent the average precision (AP) of the classification indicators. (e) Precision-recall curves of predicting interactions of protein complexes predicted by AlphaFold2 based on SpatialPPIv2, FoldDock, SpatialPPI, and average interface pLDDT score (pLDDT) and number of contacted residues. The values in brackets represent the average precision (AP) of the classification indicators. (f) Precision-recall curves of predicting interactions of protein complexes predicted by ESMFold_v1 based on SpatialPPIv2, FoldDock, SpatialPPI, and average interface pLDDT score (pLDDT) and number of contacted residues. The values in brackets represent the average precision (AP) of the classification indicators.

We used the most advanced protein structure prediction models, AlphaFold3, AlphaFold2, and ESMFold_v1, to predict the test dataset and compare the performance of SpatialPPIv2 with FoldDock and SpatialPPI, as shown in Fig. 5. Each protein pair was predicted to form a complex. Prediction methods were executed using the official default instructions. Fig. 5(a) shows the average prediction times for the three models. The prediction times of AlphaFold3 and AlphaFold2 were the sum of the total time of the complete database search of the two input proteins. The ESMFold_v1 model achieved an extremely high prediction speed by omitting the database search. Fig. 5(b) shows the distribution of the average pLDDT scores for the contact regions of the protein complex predicted by the three models. Blue represents positive samples, and orange represents the pLDDT distribution of negative samples. The horizontal axis represents the pLDDT score value of the sample, and the vertical axis corresponds to the number of samples with pLDDT distributed in the corresponding interval. Compared to AlphaFold3 and AlphaFold2, ESMFold_v1 had more negative samples, whose pLDDT was predicted to be 0. Fig. 5(c) shows the distribution of the number of contact residues as predicted by the three models. Blue represents positive samples, and orange represents negative samples. The width of each box corresponds to the distribution density of the number of contact residues represented by the corresponding y-axis for samples of this type, and the circles above and below the box represent sample cases with outlier distribution. In the prediction results of ESMFold_v1, more negative samples had no contact residues; however, the number of contact residues in the positive samples was low, resulting in some overlapping areas. AlphaFold3 showed superior separation between positive and negative samples.

Fig. 5(d), (e), and (f) show the precision-recall curves for resolving protein interactions using the protein complexes predicted by AlphaFold3, AlphaFold2, and ESMFold_v1, respectively. We compared the SpatialPPIv2, FoldDock, SpatialPPI, and pLDDT scores of the contacted residues, and the number of contact residues was directly calculated from the predicted complexes as a baseline. The baseline performance shows that using only the number of contact residues or the average pLDDT score of the contact interface can provide good performance in resolving interactions. Notably, the results of AlphaFold2 based on the number of contact residues were low, indicating that the number of contact residues of the positive and negative samples in its prediction had a large overlap. The baseline performance of ESMFold_v1 manifests as a lack of high precision and low recall. This means that some negative samples were predicted to have a high pLDDT score, and the number of contact residues was close to the highest, resulting in a failure to obtain high precision even with a large threshold.

SpatialPPIv2 showed the best classification performance, and the results predicted by AlphaFold3 and AlphaFold2 were similar to those predicted using the experimental structure. The prediction results obtained using ESMFold_v1 decreased slightly, which may be because ESMFold_v1 incorrectly predicted certain structures. The prediction results of the FoldDock model showed better separation than those of the two indicators based on the average interface pLDDT score and number of contact residues. SpatialPPI was mainly used to solve the false-positive model predicted by AlphaFold2, and it was only better than FoldDock in the prediction results based on AlphaFold2. However, SpatialPPI outperformed the baseline in the classification results based on AlphaFold3 and ESMFold. Overall, these tests demonstrate that SpatialPPIv2 remains robust when using predicted structural data, offering a promising solution for predicting interactions between proteins for which experimentally confirmed structural information is unavailable.

### 3.3 Comparing with state-of-the-art methods

Table 4 shows the performance evaluation of the state-of-the-art PPI prediction models, including SpatialPPIv2, FoldDock, Topsy-Turvy, D-script, Struct2Graph, and GNN-PPI, highlighting the differences in their abilities to predict PPIs. Performance was reported for measures of accuracy, precision, recall, F1 score, AP, AUROC, and confusion matrix. SpatialPPIv2 showed the best performance, achieving the highest AP and AUROC among all the models. The performance of FoldDock was calculated based on protein complexes predicted using AlphaFold3. FoldDock also performed well in AP and AUROC evaluations, indicating the correctness of its method for calculating pDockQ as an indicator of interactions. Similar to SpatialPPI, the main disadvantage of FoldDock is that it relies on protein complexes predicted using protein structure prediction programs. This makes it time-consuming to perform studies evaluating protein interactions and is not conducive to large-scale protein pair screening. Moreover, the threshold of 0.23, was an empirical number derived from the distribution of the prediction results based on the dataset of the original study, may be different for diverse data. For example, in our dataset, the optimal threshold was 0.21 for the pDockQ score, which achieved the highest accuracy of 0.894.

**Table 4:** Comparison of accuracy (ACC), area under receiver operating characteristic curve (AUROC), average precision (AP), F1 score, precision, recall, and confusion matrix for protein–protein interaction prediction methods.

	SpatialPPIv2	FoldDock	Struct2Graph	GNN-PPI	Topsy-Turvy	D-script
Accuracy	0.910	0.888	0.812	0.771	0.774	0.704
Precision	0.886	0.899	0.811	0.730	0.839	0.857
Recall	0.942	0.875	0.813	0.858	0.678	0.490
F1 Score	0.913	0.887	0.812	0.789	0.750	0.624
AP	0.973	0.954	0.876	0.801	0.865	0.826
AUROC	0.972	0.950	0.891	0.833	0.867	0.824

TN	527	541	486	410	522	551
FP	73	59	114	190	78	49
FN	35	75	112	85	193	306
TP	565	525	488	515	407	294

Struct2Graph achieved a balanced result for all indicators. Although it was also used to process protein structures, the two input proteins were independent of the network structure. This may lead to a lack of sufficient message exchange between the two input proteins, thereby reducing the prediction accuracy. Although GNN-PPI detected only a few false negatives, it had many false positives overall, similar to the performance of SpatialPPIv2 using one-hot encoding. This may be due to the lack of sequence processing in GNN-PPI, which leads to a lack of protein sequence context information. Struct2Graph and GNN-PPI have a lower ease of use because the trained models are not available.

The overall performances of Topsy-Turvy and D-script, two prediction programs that are completely based on sequences, are lower than those of the prediction programs based on structures. Simultaneously, both prediction methods exhibited a clear tendency to produce negative outputs. This tendency may result in higher accuracy when dealing with unbalanced datasets. However, important interactions may be overlooked when protein pairs are screened for biological experiments.

### 3.4 Large scale evaluations

To avoid potential overfitting, additional tests were performed by swapping the training and validation datasets. We trained the model using the ProtT5 language model on 2,162 pairs from the validation dataset and evaluated it on 1,167,032 pairs from the training dataset. The size of this evaluation was more than 500 times the size of the dataset of the model that was trained. The resulting confusion matrices are presented in Table 5. Overall, SpatialPPIv2 maintained an excellent performance. This validation method using a large dataset ensures a great diversity of test data during the test process and proves that SpatialPPIv2 can make correct predictions for various protein types.

**Table 5:** Confusion Matrix of evaluation on 1 million protein pairs. A=B means the two proteins are same or similar, and A≠B means the two proteins are different. Accuracy is calculated based on different data labels.

Label	Negative	Positive	Positive (A=B)	Positive (A≠B)
Predicted 0	558,236	33,835	729	33,106
Predicted 1	25,280	549,681	231,889	317,792
Accuracy	0.957	0.942	0.997	0.906

Compared with the test dataset constructed using Negatome, by comparing the positive and negative values in the confusion matrix, the classification ability of SpatialPPIv2 for protein pairs that can interact remained stable in these two evaluations, averaging an accuracy of 0.940. For protein pairs that could not interact, it was 0.957 when using the randomly matched test set and only 0.878 for non-interactive protein pairs that were experimentally verified. This indicates that experimentally verified non-interactive datasets have a high prediction difficulty. This observation was supported by the findings of Wei et al. [58] because the experimental verification was selective. Usually, only protein pairs suspected of interacting are included in experiments, whereas protein pairs that have no possibility of interaction are difficult to include in experiments. This characteristic makes it more difficult to distinguish experimentally verified non-interacting protein pairs in prediction problems than to distinguish randomly generated protein pairs.

Because there are cases where two input proteins are identical among the proteins that can interact, we separately counted the cases when the two input proteins were identical or similar, and when the two input proteins were different. The results showed that when the input proteins were the same or similar, the accuracy of the model was significantly higher than that when the input proteins were different. However, the model performed well when the input proteins were different. This demonstrated that the model did not make decisions based on whether the two input proteins were similar. The model has a higher accuracy when the input proteins are the same because most self-interacting proteins are not direct repeats. For self-interacting proteins with other binding modes such as rotational symmetry, mirror symmetry, and asymmetry, the residue intervals that may interact were calculated twice by the neural network when calculating the interaction probability. This results in proteins with higher interaction probability outputs, thereby reducing the number of false negatives.

### 3.5 Visualization of the inference process

**Fig. 6:**
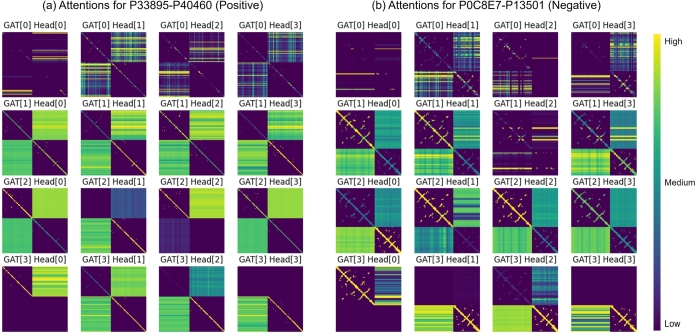
Visualization of the attention weights of the Graph Attention Network when inferring P33895-P40460 and P0C8E7-P13501. The GAT[*i*] Head[*j*] represents the attention weights of the *j*-th head of the *i*-th layer GAT. The area closer to yellow in color indicates that the attention head has a higher level of attention to that area, while the darker area means a lower level of attention. For each attention head, the attention weight on each edge is calculated based on the same trainable weight and different node and edge features. The difference in attention weight levels can reflect the importance of each edge. (a) P33895-P40460 is a protein pair that has been experimentally verified to interact. (b) P0C8E7-P13501 is a protein pair that has been experimentally verified to lack interaction.

To analyze the message-passing process in GAT, we visualized the attention weights of each attention head in GAT, as shown in Fig. 6. Fig. 6(a) shows the process of SpatialPPIv2 in inferring an example of proteins P33895 and P40460 that interact with each other [59], and Fig. 6(b) shows the process for a pair of proteins, P0C8E7 and P13501, which do not interact with each other [57]. These two protein pairs were obtained from the test dataset and inferred based on the ProtT5 language model and experimentally determined protein structures using a model trained on the training set. Both protein pairs were correctly classified using spatial SpatialPPIv2. Each figure shows the attention weights of the four attention heads of each GAT in SpatialPPIv2. Each graph shows the attention weights corresponding to the edges of the graphical representation of the input protein. The horizontal and vertical axes correspond to the sum of the lengths of the protein sequences. The color of the point in the figure represents the attention weights of the edge between the residues on the horizontal and vertical axes corresponding to the point. Brighter colors represent higher attention levels, and darker colors represent lower attention levels or no edges between the residues. The four areas in the figure represent the internal structure of input protein A, the lower right represents the internal structure of input protein B, and the upper and lower right represent the fully connected fictional edges between proteins A and B. Based on the characteristics of the GAT network structure, different levels of attention for the edge in each graph were calculated using the same trainable weight of the attention head and the difference between the node and edge features. As the trainable weights of each attention head do not change during the inference of these two examples, a comparison of these two figures shows the characteristic differences between the networks when inferencing positive and negative samples.

By comparing GAT1 in Fig. 6(a) and Fig. 6(b), especially GAT1 HEAD2, we found that the weight of the fully connected part between the two protein regions of the negative sample is significantly lower. Simultaneously, in GAT3 HEAD1 and HEAD2, the negative sample lost the connection between the two proteins, but the positive sample still affected this connection. This difference in the weights of fictional connections constitutes a difference in the output of SpatialPPIv2 between the two protein pairs. This illustrates the importance of fictional connections between residue pairs from different proteins. They build channels of message passing between the two graphs, exchanging features of the two proteins according to the adaptive attention weights. Without these connections, the two graphs would be isolated and unable to be accurately predicted.

In addition, the grid-like distribution of attention to the connection region between the two proteins confirmed that SpatialPPIv2 relies on the characteristics of the attention head for different residues and fragments to analyze protein interactions. The calculation mechanism of the attention head weight and topological structure of the protein determines that SpatialPPIv2 will not overfit specific protein sequences during the learning process. If overfitting based on sequence similarity comparison is caused by two identical proteins in the training data set, attention will be distributed in a fixed diagonal line instead of a grid distribution. Overall, the visualization of the attention weight distribution confirmed the reliability of SpatialPPIv2 in handling the relationship between residues.

## 4 Conclusion

The limitations of SpatialPPIv2 are mainly manifested in the inherent defects of machine-learning methods. Sequence similarity-based methods have advantages over machine learning methods when dealing with proteins for which similar references are available. Moreover, the errors in machine learning protein interaction prediction models are disordered. Protein pairs with high interaction potential but inability to interact, detected based on similarity, usually indicate that this protein has undergone some special mutation compared to its reference protein sequence, which makes it unable to interact. These types of data hold great research significance in biology. By contrast, errors in machine learning predictions are difficult to analyze because of the considerable number of parameters in neural networks.

SpatialPPIv2 presents significant advancements in the prediction of PPIs over its predecessor. Compared to the previous generation SpatialPPI, SpatialPPIv2 was greatly improved. SpatialPPI predicts PPIs based on the prediction results of AlphaFold Multimer, whereas SpatialPPIv2 no longer depends on the predicted complex of the structure prediction method but can run independently. Considering the huge dataset and computing requirements of the model prediction program, this improvement allows non-computer professionals to use ordinary personal computers for protein prediction, significantly improving the ease of use of this prediction method. Simultaneously, the evaluation based on the predicted structure also proved that SpatialPPIv2 can complete the prediction using the structure prediction model in the prediction of proteins without structural data. Therefore, although SpatialPPIv2 is a prediction method based on the protein structure, its application range is not limited by the availability of structural data. Regarding data processing, SpatialPPI uses a three-dimensional space with a resolution of 1 Å to represent the protein structure, whereas SpatialPPIv2 uses a graph representation based on the spatial distance to process protein structure information. This part was inspired by the distance-based residue-rendering method used in SpatialPPI. Using graphs to represent protein structures can effectively increase computational speed and reduce memory requirements, such that the model is no longer limited by protein size. SpatialPPIv2 significantly increased the size of the dataset, and the large-scale dataset ensured that the model was stable in terms of predicting various proteins. SpatialPPIv2 incorporates language models to extract features. Protein language models can provide rich contextual biochemical information without databases, allowing the model to predict interactions involving novel or engineered proteins, which are highly beneficial in synthetic biology and drug discovery.

In summary, SpatialPPIv2 achieved superior and robust performance. SpatialPPIv2 can rapidly screen for potentially interacting proteins for biological experiments. Its ease of use allows such screening to be performed rapidly and at a low cost. This type of screening can provide a direction for biological experiments and accelerate research. Its unique approach and robustness, based on predicted structures, make it a powerful tool for advancing protein interaction research, aiding drug discovery and synthetic biology, and for understanding complex biological processes.

## Declaration of generative AI and AI-assisted technologies in the writing process

No declarations were made.

## Funding

This study was financially supported by 10.13039/501100001695JST FOREST (JPMJFR216J), 10.13039/501100001691JSPS KAKENHI (JP23H03496), and 10.13039/100009619AMED BINDS (JP24ama121026).

## CRediT authorship contribution statement

**Wenxing Hu:** Writing – review & editing, Writing – original draft, Visualization, Validation, Software, Resources, Methodology, Formal analysis, Data curation, Conceptualization. **Masahito Ohue:** Writing – review & editing, Writing – original draft, Validation, Supervision, Project administration, Methodology, Investigation, Funding acquisition, Conceptualization.

## Declaration of Competing Interest

None declared.

## References

[1] Soleymani F., Paquet E., Viktor H., Michalowski W., Spinello D. (2022). Protein–protein interaction prediction with deep learning: a comprehensive review. Comput Struct Biotechnol J.

[2] Sedov I.A., Zuev Y.F. (2023). Recent advances in protein–protein interactions. Int J Mol Sci.

[3] Kewalramani N., Emili A., Crovella M. (2023). State-of-the-art computational methods to predict protein–protein interactions with high accuracy and coverage. Proteomics.

[4] Sledzieski S., Devkota K., Singh R., Cowen L., Berger B. (2023). TT3D: leveraging precomputed protein 3D sequence models to predict protein–protein interactions. Bioinformatics.

[5] Jiang Y., Wang Y., Shen L., Adjeroh D.A., Liu Z. (2022). Identification of all-against-all protein–protein interactions based on deep hash learning. BMC Bioinform.

[6] Du X., Sun S., Hu C., Yao Y., Yan Y. (2017). DeepPPI: boosting prediction of protein–protein interactions with deep neural networks. J Chem Inf Model.

[7] Sledzieski S., Singh R., Cowen L., Berger B. (2021). D-SCRIPT translates genome to phenome with sequence-based, structure-aware, genome-scale predictions of protein-protein interactions. Cell Syst.

[8] Singh R., Devkota K., Sledzieski S., Berger B., Cowen L. (2022). Topsy-Turvy: integrating a global view into sequence-based PPI prediction. Bioinformatics.

[9] Bhat P., Patil N. (2023). An exhaustive review of computational prediction techniques for PPI sites, protein locations, and protein functions. Netw Model Anal Health Inform Bioinforma.

[10] Yuan Q., Chen J., Zhao H., Zhou Y., Yang Y. (2021). Structure-aware protein–protein interaction site prediction using deep graph convolutional network. Bioinformatics.

[11] Gao Z., Jiang C., Zhang J., Jiang X., Li L. (2023). Hierarchical graph learning for protein–protein interaction. Nat Commun.

[12] Song B., Luo X., Luo X., Liu Y., Niu Z. (2021). Learning spatial structures of proteins improves protein–protein interaction prediction. Brief Bioinform.

[13] Lv G., Hu Z., Bi Y., Zhang S. (2021). Proceedings of the thirtieth international joint conference on artificial intelligence (IJCAI-21).

[14] Baranwal M., Magner A., Elvati P., Saldinger J., Violi A. (2019). A deep learning architecture for metabolic pathway prediction. Bioinformatics.

[15] Zhou H., Wang W., Jin J., Zheng Z., Zhou B. (2022). Graph neural network for protein–protein interaction prediction: a comparative study. Molecules.

[16] Dunham B., Ganapathiraju M.K. (2021). Benchmark evaluation of protein–protein interaction prediction algorithms. Molecules.

[17] Jumper J., Evans R., Pritzel A., Green T., Figurnov M. (2021). Highly accurate protein structure prediction with AlphaFold. Nature.

[18] Moult J., Schwede T., Kryshtafovych A., Tramontano A., Fidelis K. (2021). Critical assessment of methods of protein structure prediction (CASP)—progress and new directions in round XIV. Proteins, Struct Funct Bioinform.

[19] Abramson J., Adler J., Dunger J., Evans R., Green T. (2024). Accurate structure prediction of biomolecular interactions with AlphaFold 3. Nature.

[20] Lin Z., Akin H., Rao R., Hie B., Zhu Z. (2023). Evolutionary-scale prediction of atomic-level protein structure with a language model. Science.

[21] Bryant P., Pozzati G., Elofsson A. (2022). Improved prediction of protein-protein interactions using AlphaFold2. Nat Commun.

[22] Hu W., Ohue M. (2024). SpatialPPI: three-dimensional space protein-protein interaction prediction with AlphaFold multimer. Comput Struct Biotechnol J.

[23] Evans R, O'Neill M, Pritzel A, Antropova N, Senior A, et al. Protein complex prediction with AlphaFold-Multimer. bioRxiv 2021.

[24] Kovtun D, Akdel M, Goncearenco A, Zhou G, Holt G, et al. PINDER: the protein interaction dataset and evaluation resource. bioRxiv 2024.

[25] Morehead A., Chen C., Sedova A., Cheng J. (2023). DIPS-plus: the enhanced database of interacting protein structures for interface prediction. Sci Data.

[26] Mi Y., Marcu S., Yallapragada V.V.B., Tabirca S. (2024). ProteinFlow: an advanced framework for feature engineering in protein data analysis. Biotechnol Bioeng.

[27] Bushuiev A., Bushuiev R., Filkin A., Kouba P., Gabrielova M. Learning to design protein-protein interactions with enhanced generalization. https://openreview.net/forum?id=xcMmebCT7s.

[28] Berman H.M., Battistuz T., Bhat T.N., Bluhm W.F., Bourne P.E. (2002). The protein data bank. Acta Crystallogr, D Biol Crystallogr.

[29] Tunyasuvunakool K., Adler J., Wu Z., Green T., Zielinski M. (2021). Highly accurate protein structure prediction for the human proteome. Nature.

[30] Nath A., Leier A. (2020). Improved cytokine–receptor interaction prediction by exploiting the negative sample space. BMC Bioinform.

[31] Trabuco L.G., Betts M.J., Russell R.B. (2012). Negative protein–protein interaction datasets derived from large-scale two-hybrid experiments. Methods.

[32] Blohm P., Frishman G., Smialowski P., Goebels F., Wachinger B. (2013). Negatome 2.0: a database of non-interacting proteins derived by literature mining, manual annotation and protein structure analysis. Nucleic Acids Res.

[33] Goldberg D.S., Roth F.P. (2003). Assessing experimentally derived interactions in a small world. Proc Natl Acad Sci USA.

[34] Elnaggar A., Heinzinger M., Dallago C., Rehawi G., Wang Y. (2021). ProtTrans: toward understanding the language of life through self-supervised learning. IEEE Trans Pattern Anal Mach Intell.

[35] Devlin J., Chang M., Lee K., Toutanova K. (2019). Proceedings of NAACL-HLT 2019.

[36] Rives A., Meier J., Sercu T., Goyal S., Lin Z. (2021). Biological structure and function emerge from scaling unsupervised learning to 250 million protein sequences. Proc Natl Acad Sci USA.

[37] Wu F., Wu L., Radev D., Xu J., Li S.Z. (2023). Integration of pre-trained protein language models into geometric deep learning networks. Commun Biol.

[38] Jha K., Karmakar S., Saha S. (2023). Graph-BERT and language model-based framework for protein–protein interaction identification. Sci Rep.

[39] Gao M., Skolnick J. (2010). iAlign: a method for the structural comparison of protein–protein interfaces. Bioinformatics.

[40] Adhikari B., Cheng J. (2016). Protein residue contacts and prediction methods. Methods Mol Biol.

[41] Doerr A. (2009). The importance of being negative. Nat Methods.

[42] Hermjakob H., Montecchi-Palazzi L., Lewington C., Mudali S., Kerrien S. (2003). IntAct: an open source molecular interaction database. Nucleic Acids Res.

[43] Stark C. (2005). BioGRID: a general repository for interaction datasets. Nucleic Acids Res.

[44] (2023). https://huggingface.co/Rostlab/prot_bert.

[45] Gao Q., Zhang C., Li M., Yu T. (2024). Protein–protein interaction prediction model based on ProtBERT-BiGRU-attention. J Comput Biol.

[46] (2023). https://huggingface.co/Rostlab/prot_t5_xl_uniref50.

[47] Facebookresearch (2022). GitHub - facebookresearch/esm: evolutionary scale modeling (esm): pretrained language models for proteins. https://github.com/facebookresearch/esm.

[48] Apweiler R., Bairoch A., Wu C.H., Barker W.C., Boeckmann B. (2003). UniProt: the universal protein knowledgebase. Nucleic Acids Res.

[49] Ferruz N., Schmidt S., Höcker B. (2022). ProtGPT2 is a deep unsupervised language model for protein design. Nat Commun.

[50] Huang Y., Wuchty S., Zhou Y., Zhang Z. (2023). SGPPI: structure-aware prediction of protein–protein interactions in rigorous conditions with graph convolutional network. Brief Bioinform.

[51] Veličković P., Cucurull G., Casanova A., Romero A., Liò P., Bengio Y. Graph attention networks. https://openreview.net/forum?id=rJXMpikCZ.

[52] Jha K., Saha S., Singh H. (2022). Prediction of protein–protein interaction using graph neural networks. Sci Rep.

[53] Dwivedi V.P., Joshi C.K., Luu A.T., Laurent T., Bengio Y., Bresson X. (2022). Benchmarking graph neural networks. J Mach Learn Res.

[54] Rao R., Meier J., Sercu T., Ovchinnikov S., Rives A. Transformer protein language models are unsupervised structure learners. https://openreview.net/forum?id=fylclEqgvgd.

[55] (2022). https://gitlab.com/ElofssonLab/FoldDock/-/blob/main/src/pdockq.py.

[56] Wu Y., He K. (2018). Proceedings of the european conference on computer vision (ECCV).

[57] Dias J.M., Losberger C., Déruaz M., Power C.A., Proudfoot A.E.I. (2009). Structural basis of chemokine sequestration by a tick chemokine binding protein: the crystal structure of the complex between evasin-1 and CCL3. PLoS ONE.

[58] Wei L., Xing P., Zeng J., Chen J., Su R. (2017). Improved prediction of protein–protein interactions using novel negative samples, features, and an ensemble classifier. Artif Intell Med.

[59] Newman J.R.S., Wolf E., Kim P.S. (2000). A computationally directed screen identifying interacting coiled coils from Saccharomyces cerevisiae. Proc Natl Acad Sci USA.

